# Using gene expression data to identify causal pathways between genotype and phenotype in a complex disease: application to Genetic Analysis Workshop 19

**DOI:** 10.1186/s12919-016-0009-x

**Published:** 2016-10-18

**Authors:** Holly F. Ainsworth, Heather J. Cordell

**Affiliations:** Institute of Genetic Medicine, Newcastle University, International Centre for Life, Central Parkway, Newcastle upon Tyne, NE1 3BZ UK

## Abstract

We explore causal relationships between genotype, gene expression and phenotype in the Genetic Analysis Workshop 19 data. We compare the use of structural equation modeling and a Bayesian unified framework approach to infer the most likely causal models that gave rise to the data. Testing an exhaustive set of causal relationships between each single-nucleotide polymorphism, gene expression probe, and phenotype would be computationally infeasible, thus a filtering step is required. In addition to filtering based on pairwise associations, we consider weighted gene correlation network analysis as a method of clustering genes with similar function into a small number of modules. These modules capture the key functional mechanisms of genes while greatly reducing the number of relationships to test for in causal modeling.

## Background

Even though genome-wide association studies (GWAS) have been very successful over the past decade at identifying genetic variants associated with disease, the mechanism underlying these associations is generally not known. It is hoped that gene expression data could provide the missing link between genotype and phenotype. We are interested in exploring causal relationships between genotype data, gene expression data, and phenotype.

**Fig. 1:**
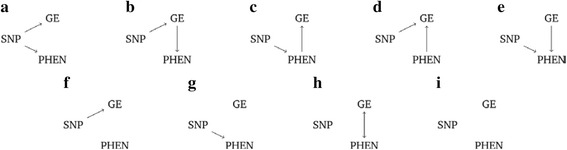
Possible causal models **a**–**i** as described in the text. GE, gene expression; PHEN, phenotype. The absence of an arrow between variables represents the lack of a causal relationship

There exist many techniques for causal analysis which could be applied to the Genetic Analysis Workshop 19 (GAW19) data. We choose to focus on structural equation modeling (SEM) and a Bayesian unified framework (BUF) approach [1]. For both of these methods, graphical models provide a natural framework for describing causal relationships. Suppose we have data from a single single-nucleotide polymorphism (SNP), gene expression measurements from a single probe, and data on a phenotype of interest. Some possible causal models are illustrated in Fig. 1. In this framework, nodes represent measured variables and the presence of a directed arrow between two nodes implies a causal link.

In Fig. 1 causal scenario (a), the SNP affects both gene expression (GE) and phenotype (PHEN) independently. Scenario (b) represents the situation where the phenotype is indirectly influenced by the SNP through gene expression. Conversely, in scenario (c), gene expression is indirectly influenced by the SNP via the phenotype. Scenarios (d) to (i) depict further possible causal scenarios; the absence of an arrow between variables represents the lack of a causal relationship.

## Methods

We performed quality control (QC) on the GAW19 GWAS (SNP) data [2] using standard procedures outlined in Anderson et al [3]. Individual level QC resulted in 4 individuals being excluded as a result of no genotype data being available. A further individual was excluded because of outlying ethnicity. SNP level QC removed 43,986 SNPs with low frequency (minor allele frequency <1 %) and 109 SNPs with high rates of missingness.

Gene expression measurements as described in Göring et al [4] are available on 638 of the individuals for whom we have GWAS data. For the causal analysis we decided to focus on the real phenotypes systolic (SBP) and diastolic blood pressure (DBP) and we only included individuals for whom we have both GWAS and gene expression data. For each of the phenotypes, we adjusted for covariates using linear regression and took the average of the residuals over the different time points within an individual as our final phenotype. Covariates in the models included age, medication status, smoking status, and (for DBP) age squared. This replicates the analysis performed by Eu-Ahsunthornwattana et al [5].

### Filtering

Rather than conducting causal analysis for every possible trio of SNP, gene expression, and phenotype measurements, a filtering step was undertaken to identify potentially interesting trios (see, eg, Liu et al [6] and Shin et al [7]). First, association analysis was conducted to identify gene expression probes that were correlated with the phenotypes. This was done separately for SBP and DBP using linear regression with the gene expression measurements corrected for sex. For gene expression probes that showed an association with phenotype, a genome-wide association scan was carried out with gene expression as the phenotype. This analysis was carried out using the Factored Spectrally Transformed Linear Mixed Model (FaST-LMM) software [8], which models relatedness between individuals as estimated through genome-wide SNP data. SNPs that showed an association with the gene expression probes were retained. The end result of this filtering step is a significantly reduced number of trios of SNP/gene expression/phenotype variables on which to perform causal analysis.

### Weighted gene correlation network analysis

An alternative to the above filtering step is to use the weighted gene correlation network analysis (WGCNA) approach [9], which allows us to group genes into a relatively small number of modules (clusters). These modules contain sets of highly correlated genes based on their gene expression measurements. Each module can be summarized by an eigengene that can be taken forward to be used in further analyses. This process is described in Ghazalpour et al [10].

### Causal modeling

Causal modeling was performed for all trios of SNP, gene expression, and phenotype that remained following filtering, using the 2 methods outlined below.

### Structural equation modeling (SEM)

SEM is a regression-based approach to causal modeling. A system of linear equations can be constructed based on the relationships between nodes in the graphical model. The parameters in the model can be estimated using maximum likelihood and the fit of the model evaluated using appropriate statistical tests. When more than 1 causal model is tested, the one with the lowest Akaike information criterion (AIC) can be thought of as representing the most plausible underlying causal mechanism.

A selection of possible causal models which can be tested in the SEM framework are depicted in Fig. 1. Note that biologically implausible models (ie, any model in which the SNP is causally affected by another variable) have been omitted.

### Bayesian unified framework (BUF)

The BUF [1] is a flexible approach that can be used for univariable and multivariable testing. The approach has its foundations in Bayesian model comparison and model averaging. This approach partitions variables in the model into subsets $\gamma =\left(U,D,I\right)$ with respect to a predictor variable, in our case the SNP. The variables in *U* are unassociated with the SNP, the variables in *D* are directly associated with the SNP and variables in *I* are indirectly associated with the SNP. For each possible partition, a Bayes’ factor is computed; the model with the highest Bayes’ factor can be interpreted as the one that best fits the data. For example, if the model with the highest Bayes’ factor classifies gene expression as directly associated with the SNP (D) and phenotype as indirectly associated with the SNP (I) then this is equivalent to model (b) in Fig. 1.

## Results

**Fig. 2:**
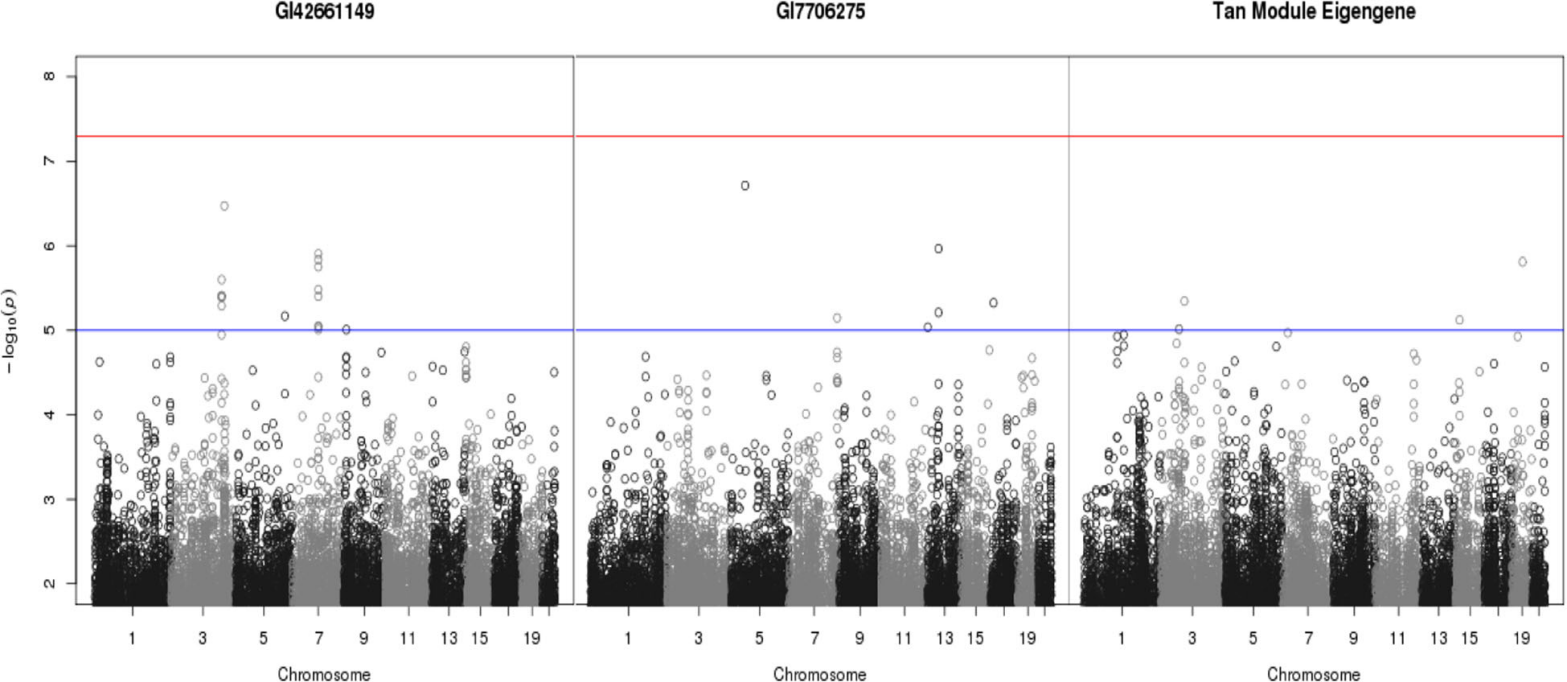
GWAS results using gene expression (left and center) or module eigengene (right) as phenotype

The first filtering step identified 2 gene expression probes that showed marginally significant correlation (−log_10_ *p* value >5) with SBP. These were GI42661149, for which no further information was given in the map file, and GI7706275, which lies in the gene *TPPP3* on chromosome 16 at coordinates 67423801 bp to 67423850 bp. Two GWAS were performed using these gene expression probes as the phenotype (Fig. 2). All SNPs that showed marginal significance (−log_10_ *p* value >5) with either gene expression probe (a total of 24 SNPs) were taken forward to be used in causal analysis.

Table 1 shows the results of causal analysis. In 16 cases, both SEM and BUF agreed that the causal relationships should be classified as model (b). In the other 8 cases, SEM and BUF disagreed. For example, in 3 cases the causal relationships were classed as model (d) by SEM and as model (f) by BUF. Model (d) represents the scenario where gene expression is influenced independently by both SNP and phenotype, while model (f) represents the scenario whereby gene expression is directly associated with SNP and phenotype is unassociated with SNP. Note that (d) is not currently tested for by BUF, and (f) is not tested for by SEM.

**Table 1:** Results of causal analysis using gene expression probes

SNP				Preferred model	
SNP name	Chromosome (bp position)	Gene expression probe	Phenotype	SEM	BUF
rs9869956	3 (155164342)	GI42661149	SBP	(b)	(b)
rs11709568	3 (155177333)	GI42661149	SBP	(b)	(b)
rs6440993	3 (155180174)	GI42661149	SBP	(b)	(b)
rs9829532	3 (155181831)	GI42661149	SBP	(b)	(b)
rs7618495	3 (155184048)	GI42661149	SBP	(b)	(a)
rs4680185	3 (155213002)	GI42661149	SBP	(b)	(b)
rs822711	3 (165003805)	GI42661149	SBP	(b)	(b)
rs4921240	5 (159327355)	GI42661149	SBP	(b)	(b)
rs9640732	7 (78320848)	GI42661149	SBP	(b)	(b)
rs13246026	7 (78331066)	GI42661149	SBP	(b)	(b)
rs7793494	7 (78344389)	GI42661149	SBP	(b)	(b)
rs11768116	7 (78345207)	GI42661149	SBP	(b)	(b)
rs13242288	7 (78345298)	GI42661149	SBP	(b)	(b)
rs7779874	7 (78346879)	GI42661149	SBP	(d)	(b)
rs757395	7 (78356891)	GI42661149	SBP	(b)	(f)
rs1888238	7 (78390300)	GI42661149	SBP	(b)	(b)
rs13308578	7 (78408559)	GI42661149	SBP	(b)	(b)
rs7038267	9 (4821348)	GI42661149	SBP	(d)	(f)
rs34868670	5 (40237843)	GI7706275	SBP	(d)	(a)
rs10246727	7 (152876977)	GI7706275	SBP	(b)	(b)
rs2451078	13 (20098289)	GI7706275	SBP	(d)	(a)
rs1570621	13 (47170118)	GI7706275	SBP	(d)	(f)
rs4942556	13 (47174585)	GI7706275	SBP	(b)	(b)
rs1105813	17 (7721542)	GI7706275	SBP	(d)	(f)

### Weighted gene correlation network analysis method

**Fig. 3:**
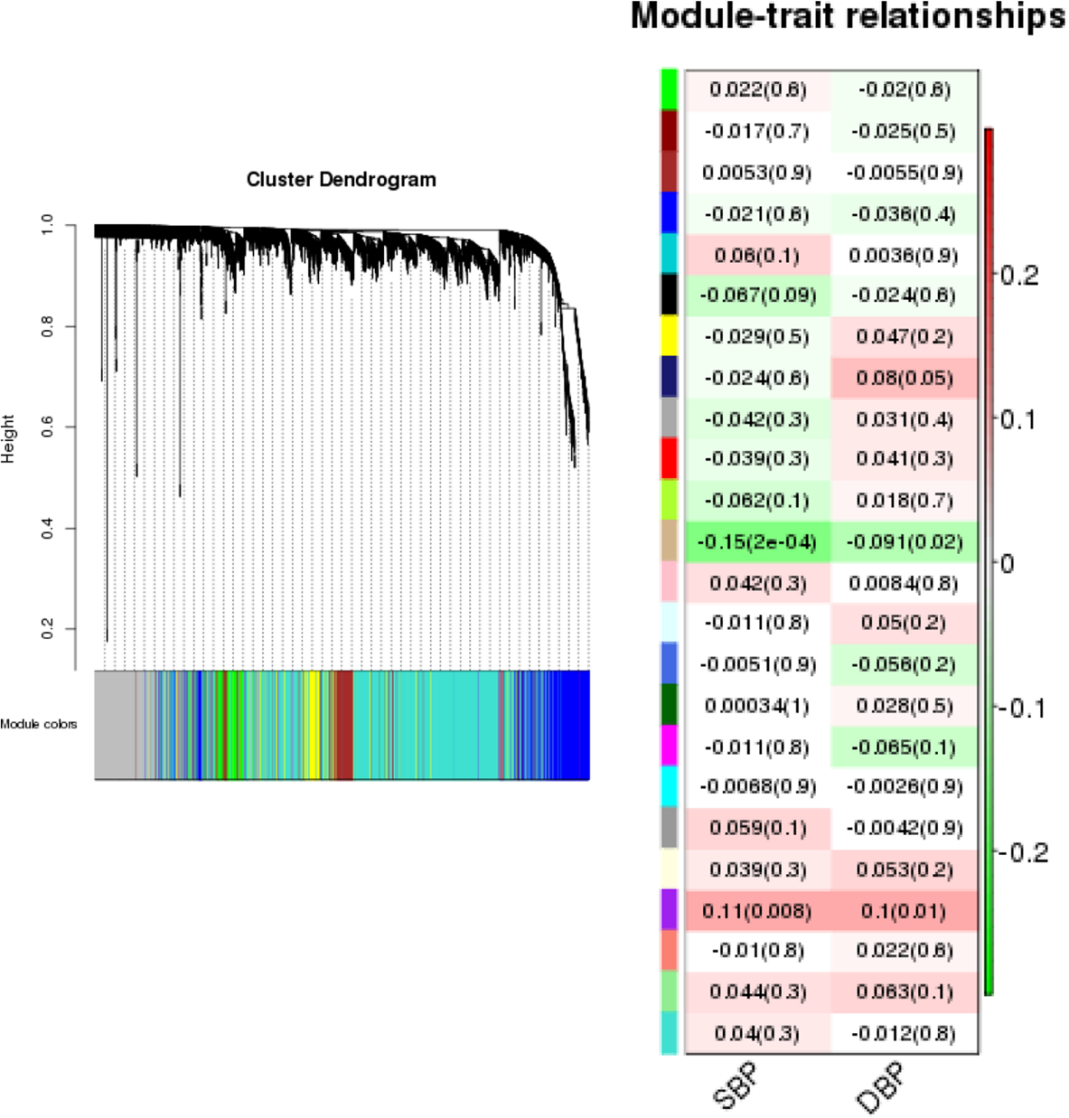
Results from WCGNA. *Left:* Dendrogram showing clustering of gene expression probes into different colored modules. *Right:* Correlation between phenotypes and module eigengenes for different colored modules (with *p* values in parentheses)

The WGCNA package in R [9] was used to cluster gene expression measurements into modules (typically identified by different colors). A total of 24 modules were identified; a dendrogram depicting the results of this clustering can be viewed in Fig. 3 (left-hand plot). For each module, the gene expression profile is summarized by a module eigengene. The correlation between the module eigengene and the phenotypes (SBP and DBP) was tested for each module (Fig. 3, right-hand plot). There was 1 module/phenotype pairing (the tan colored module and SBP) that was statistically significant (*p* value = 0.0002) using a Bonferroni correction to set the appropriate threshold to account for multiple testing.

A GWAS was performed using the tan module eigengene as the phenotype (see right-hand plot of Fig. 2). This returned 5 SNPs that showed modest significance with the phenotype. These SNPs were taken forward to be used in causal analysis and Table 2 shows the results. As previously, in the majority of cases, both SEM and BUF suggested that the inferred causal relationship should be (b). There was also 1 case where SEM suggested (b) and BUF suggested (a).

**Table 2:** Results of causal analysis using WGCNA

				Preferred model	
SNP name	Chromosome(bp position)	Module eigengene	Phenotype	SEM	BUF
rs9844757	3 (49343601)	Tan	SBP	(b)	(b)
rs34823813	3 (49749976)	Tan	SBP	(b)	(b)
rs1374678	3 (63083408)	Tan	SBP	(b)	(b)
rs2568532	15 (34387365)	Tan	SBP	(b)	(b)
rs12983427	19 (34307901)	Tan	SBP	(b)	(a)

## Discussion

Two methods for causal inference were implemented to identify causal pathways between SNP, gene expression, and phenotype. Attempting causal analysis without a stringent filtering process would not be possible and WGCNA provides a convenient tool in this process. SEM and BUF were generally in agreement in their assessment of the underlying causal model, although the true underlying causal mechanisms are not known, so these inferences cannot be validated. However, when SEM and BUF disagreed, it must be noted that the difference in Bayes’ factors between competing models in the BUF approach was very small.

SEM has the advantage of allowing models such as model (d), whereby the phenotype affects gene expression independently from the SNP, to be tested; in the BUF approach, this relationship is not routinely tested. Despite this, the BUF approach is more flexible than SEM because in BUF, an exhaustive set of partitions for variables is automatically tested for; this could be especially useful if there were a large number of variables in the model.

A direct comparison between filtering and WGCNA is not straightforward; however, we note that there are no overlapping gene expression probes between the tan module and the probes obtained using filtering. WGCNA takes approximately 1.5 hours to run and the tan module eigengene accounts for 2.1 % of the variation in SBP. However, filtering takes only a few minutes and the 2 gene expression probes identified account for 3.6 % and 2.8 %, respectively, of the variation in SBP.

For our purposes, both SEM and BUF are very quick to perform, with both methods taking no more than a couple of seconds. However, it must be noted that when we consider the computational cost of our methods, we are focusing on the very simple case where we have only 3 variables. Where many more variables are present, we suggest more investigation is required to assess the scalability of SEM, BUF and WGCNA.

We also considered performing causal analysis on the simulated phenotypes in the GAW19 data set. However, at the first filtering step we failed to recover any significant associations between phenotype and gene expression probes located in genes that featured in the true underlying simulation model. Consequently, we did not proceed any further with causal analysis.

## Conclusions

Two methods for causal inference were implemented to identify causal pathways between SNP, gene expression, and phenotype on the reduced number of trios. The methods displayed reasonably good concordance with the same causal model being identified the majority of the time. Both methods were easy and quick to implement for the simple cases we considered in this analysis. However, using these methods for an analysis with many more variables would require careful thought.
